# A survey of engagement and competence levels in interventions and activities in a community mental health workforce in England

**DOI:** 10.1186/1472-6963-11-352

**Published:** 2011-12-29

**Authors:** Linda Lang, Sophie Orton, David Sallah, Teresa Hewitt-Moran, Dongmei Zhang, Sean Cullen, Sheila Dixon, Brian Bell, David Bell, Lesley Meeson, Ruoling Chen

**Affiliations:** 1 School of Health and Wellbeing, University of Wolverhampton, Nursery Street Wolverhampton WV1 1AD, UK; 2 Leicester Partnership NHS Trust, George Hine House, Towers Hospital, Gipsy Lane, LE5 0TD, UK; 3 West Midlands Workforce Deanery, West Midlands Strategic Health Authority, St. Chads Court, 213 Hagley Road, Birmingham B16 9RG, UK; 4 Department of Health Services Administration, Anhui Medical University, 81 Meishan Road, Hefei 230032, China; 5 Coventry & Warwickshire Partnership Trust, Wisons Lane, Coventry CV6 6NY, UK; 6 Department of Primary Care and Public Health Sciences, King's College London, 42 Weston Street, London SE1 3QD, UK

## Abstract

**Background:**

National Health Service (NHS) mental health workforce configuration is at the heart of successful delivery, and providers are advised to produce professional development strategies. Recent policy changes in England have sharpened the focus on competency based role development. We determined levels of intervention activities, engagement and competence and their influencing factors in a community-setting mental health workforce.

**Methods:**

Using a modified questionnaire based on the Yorkshire Care Pathways Model we investigated 153 mental health staff working in Coventry and Warwickshire NHS Trust. A median score of competence was computed across 10 cluster activities. Low engagement and competence levels were examined in a logistic regression model.

**Results:**

In 220 activities, Monitoring risk was the highest rate of engagement (97.6%) and Group psychological therapy/Art/Drama therapy was the lowest engagement (3.6%). The median competence level based on all activities was 3.95 (proficient). There were significant differences in the competence level among professional groups; non-qualified support group (3.00 for competent), Counsellor/Psychologist/Therapist (3.38), Occupational therapists (3.76), Nurses (4.01), Medical staff (4.05), Social workers (4.25) and Psychologists (4.62 for proficient/expert). These levels varied with activity clusters; the lowest level was for Counsellor/Psychologist/Therapist in the accommodation activity (1.44 novice/advance beginner) and the highest for Occupational therapists in personal activity (4.94 expert). In a multivariate analysis, low competence was significantly related to non-qualified community support professions, late time of obtaining first qualification, more frequencies of clinical training, and training of cognitive behavioural therapy. The associations were similar in the analysis for 10 activity clusters respectively.

**Conclusions:**

There was a reasonable competence level in the community-setting mental health workforce, but competence varied with professional groups and cluster activities. New staff and other non-qualified support professions need to receive efficient training, and the training content is more important than frequency to increase level of competence.

## Background

The National Service Framework (NSF) for Mental Health in England [1] has recognised that workforce configuration will be at the heart of successful delivery, and providers are advised to produce professional development strategies as the balance of existing skills will need to be adjusted in some areas in order to address a shortage of skills in others. The National Health Service (NHS) Plan [2] strengthened the messages delivered in the NSF, announcing major investment in new models of service: crisis resolution, assertive outreach, and early intervention in psychosis teams, and new roles. Whilst the NSF made specific references to the perceived challenges for professions, the NHS Plan with its new roles and new team models with a specific multidisciplinary focus did not. The message was clear, however, that not only were professions being asked to work in more prescribed multidisciplinary and interdisciplinary models, but also that government policy was looking beyond professional roles to new roles based on competencies.

More recent English Government policy changes have sharpened the focus on competency based role development. In Lord Darzi's final report of the Next Stage Review, *High Quality Care for All *[3], the government outlined a commitment to develop a common currency system for contracting for mental health services. The Department of Health circular letter [1] announced that this currency system would be available by March 2011, and would be based upon the Care Pathways and Packages methodology known as the Yorkshire Care Pathways Model (YCPM), that had been developed by six mental health trusts in the Yorkshire and Humber and North East regions of England.

The Strategic Health Authority of the West Midlands region of England decided to adopt the YCPM early, utilising the Commissioning for Quality and Innovation Payment Framework [4] to incentivise and encourage provider organisations to allocate all new referrals to the pathway clusters by April 2010. Early development of the currency system was driven utilising tools developed by the productivity programme utilising the YCPM [5]. The differentiation between need and demand in health care commissioning and planning is recognised as challenging. The model attempts to provide a validated clinical decision-making support tool, for the classification of conditions and relevant therapeutic interventions. It is argued that this benefits both service users and health care providers by informing more rational systematic service planning and reducing inter-professional variations. The YCPM allocates service users into care clusters dependent upon their needs or characteristics. By outlining service activities within each cluster, care can be standardised through systematic linking of resources and better identification of service user needs [5]. The YCPM is specifically designed to support multi-professional working; however, there was no clear indication of the competencies required within the workforce to deliver these interventions, or how they may be best organised. In this study, we carried out a questionnaire survey in a community-setting mental health multi-professional workforce in West Midlands region of England, to investigate activities/interventions, engagement and levels of competency and their determinants.

## Method

### Questionnaire

We employed a modified 'Therapeutic Interventions and Activities Audit Questionnaire' from the YCPM [5] to assess the engagement and competency level. This had the advantage of enabling wide distribution to staff.

The questionnaire was designed by Coventry and Warwickshire Partnership NHS Trust based upon the Care Packages and Pathways Model devised by South West Yorkshire Mental Health Trust. The model has needs-based care clusters related to service users' symptoms, symptom severity, complexity of disorder and chronicity of disorder [5].

It contained 220 items, each one being a specific care activity as identified in the YCP model. The items were broadly categorised into the 10 major care clusters; (1) Assessment activities, (2) Monitoring activities, (3) Enabling activities, (4) Therapeutic interventions, (5) Role support activities, (6) Family/carer interventions, (7) Accommodation, (8) Care co-ordination, (9) Social participation, and (10) Personal and professional care management capabilities. Each care cluster was further subcategorised within the questionnaire to form 47 distinct areas of care; therefore the overarching assessment activities care cluster was further broken down into assessment of mental state, assessment of role functioning, assessment of risk and so on.

Frequency of engagement for each individual care activity was determined by asking participants to state whether they were '*regularly and actively engaged in*' specified activities. If they stated that they were, then they were asked to rate their competence level for this activity. The questionnaire adopted Benner's From Novice to Expert scale [6]; participants rated their competence level on 1 - 5 likert scales ranging from (1) novice, (2) advanced beginner, (3) competent, (4) proficient and (5) expert.

To examine determinants of engagement and competency level, we included participants' demography and other variables in the questionnaire, such as time of obtaining the first degree. Two focus groups were conducted with a small sample of Trust staff after the data gathered from the questionnaires had been initially analysed, in order to triangulate findings and enrich interpretation (Mental Health Clinical Care Pathways Project - A final report to West Midlands Strategic Health Authority 2010).

### Participants

We selected the Coventry and Warwickshire NHS Trust for our study field as this trust had adopted the use of the Yorkshire Care Pathway in the previous year. We targeted all community-based adult mental health teams (524 staff), including three older adult teams, one specialist older adult and three specialist adult teams.

In September to December 2009 we sent the questionnaire with a cover letter to 387 staff to complete and post back to the research team. One hundred and fifty three staff members returned their completed questionnaires, with a response rate of 39.5%. The range of occupational groups among the participants included: qualified nurses (57.5%), occupational therapists (9.9%), psychologists (7.2%), medical staff (2.6%), counsellor/psychotherapist/therapist (3.9%), social worker (5.2%), and non-qualified support staff (13.2%) (including support, time and recovery workers (STR), community supporters and nurses).

### Statistical analysis

We analysed data of engagement and competence levels in total, and in 10 therapeutic activity clusters respectively. We also examined data of 47 subheading clusters where needed. We calculated a total score of engagement activities based on the participant's actual engagement, and accordingly computed the score for each cluster through the total number of activities within each cluster engaged in by the participant.

We added the competence level (score) from each activity in which the participant engaged in total and in their 10 therapeutic activity clusters, and calculated an average score for the total activity and 10 clusters (i.e., the added score was divided by the number of activities engaged).

We examined the distribution of engagement and competence levels among occupational groups and other demographic factors using Chi-square or non-parametric K-W tests for categorical or continuous variables. A Spearman correlation was used to investigate a relationship between the number of engagement activities and competence levels. We employed a logistic regression model to investigate associations of low engagement or low competence (cut-off point at their median level) with determinants factors.

All analyses were performed using the SPSS statistical package (Windows version 16.0; SPSS Inc., Chicago, Illinois).

## Results

Among 220 activities, the highest rate of engagement activities among participants was Monitoring risk at 97.6% and the lowest engagement was Group psychological therapy/art/drama therapy at 3.6%. Table 1 shows the 8 highest and 8 lowest engagement activities.

**Table 1:** Most and least commonly engaged in activities in 153 staff in the adult mental health teams in Coventry and Warwickshire NHS Mental Health Trust

Title of Activity	%
***Most commonly engaged in activities***	
Monitoring activities: Monitoring risk	97.6
Monitoring activities: Level of engagement/Therapeutic relationship	97.0
Monitoring activities: Mental state	96.4
Role support activities: Informal Counselling, listening	96.4
Assessment activities: Assessment of mental state, general observation: appearance, psychomotor activity, attitude.	95.8
Assessment activities: Assessment of mental state, thought processes	95.8
Assessment activities: Assessment of mental state, speech	95.2
Enabling activities: Assertive engagement, positive engagement to build a relationship of trust	95.2
	
***Least commonly engaged in activities***	
Role support activities: Social Support, Music	9.6
Role support activities: Social Support, Cooking	9.6
Therapeutic interventions: Medical treatments, Assisting in the administration & recovery from ECT	7.8
Therapeutic interventions: Group Psychological Therapy, Psychodynamic therapy	7.8
Therapeutic interventions: Structured Psychological Therapy, Art/drama therapy	7.2
Therapeutic interventions: Group Psychological Therapy, Dialectic Behaviour Therapy	6.0
Therapeutic interventions: Medical treatments, Prescribing ECT	4.8
Therapeutic interventions: Group Psychological Therapy, Art/drama therapy	3.6

In the analysis of 47 subheading clusters, we found that *Monitoring activities *(including mental state, risk, physical health and wellbeing), *Enabling activities - assertive engagement *(including positive engagement to build a relationship of trust and positive engagement to be reliable) and *Role support activities, informal counselling *(including listening, empathising, reflecting) were the three most common activities with 94.8% of the sampled staff reporting to engage in them. The three least commonly engaged in activities were *Role support activities - social support *(including accompanying service users to the cinema, sport, art and social clubs) at 12.4%, *Assessment activities - diagnosis *at 23.5% and *Assessment activities - dementia care mapping *at 24.2%.

Table 2 illustrates the median competency levels in the total, and in the 10 activity clusters across the occupational groups. The median competence level for all activities was 3.95. It varied with activity clusters; the lowest competence level (3.40) was for therapeutic interventions and the highest (4.08) for care co-ordination activities.

**Table 2:** Competency levels for total engagement activities and the 10 activity cluster across occupational groups

	Activity Cluster										
	
Occupational group	Total N = 153	Assessment N = 150	Monitoring N = 150	Enabling N = 151	Therapeutic N = 142	Role N = 147	Family N = 137	Accommodation N = 90	Care N = 132	Social N = 115	Personal N = 141
All participants (n = 153)	**3.95**	3.88	4.00	3.95	3.40	4.00	4.00	3.83	4.08	3.88	4.00
Non qualified support (community support, nursing non qualified, STR worker) (n = 20)	**3.00 *(1)^†^***	3.00 *(1)*	3.00 ***(1)***	3.00 ***(1)***	3.00 ***(1)***	3.15 ***(2)***	3.00 ***(2.5)***	3.17 ***(3)***	3.00 ***(1)***	3.00 ***(2)***	3.00 ***(1)***
											
Counsellor, psychotherapist, therapist (n = 6)	**3.38 *(2)***	3.30 ***(2)***	4.20 ***(4)***	3.24 ***(2)***	3.45 ***(4)***	3.50 ***(3)***	2.90 ***(1)***	N/A	3.71 ***(2)***	3.31 ***(3)***	3.31 ***(2)***
											
Medical (n = 4)	**4.05 *(5)***	4.40 ***(6)***	4.63 ***(6)***	3.50 ***(3)***	3.58 ***(6)***	3.13 ***(1)***	3.00 ***(2.5)***	1.44 ***(1)***	4.00 ***(4)***	2.60 ***(1)***	4.09 ***(5)***
											
Nursing qualified (n = 88)	**4.01 *(4)***	4.00 ***(4)***	4.00 ***(2.5)***	4.00 ***(5)***	3.52 ***(5)***	4.22 ***(4)***	4.00 ***(5)***	3.88 ***(5)***	4.13 ***(5)***	4.00 ***(5.5)***	4.00 ***(3.5)***
											
Occupational therapists (n = 15)	**3.76 *(3)***	3.71 ***(3)***	4.00 ***(2.5)***	3.73 ***(4)***	3.32 ***(3)***	4.25 ***(5)***	3.86 ***(4)***	3.44 ***(4)***	3.90 ***(3)***	3.90 ***(4)***	4.00 ***(3.5)***
											
Psychologist (n = 11)	**4.62 *(7)***	4.51 *(7)*	5.00 ***(7)***	4.65 ***(7)***	4.81 ***(7)***	5.00 ***(7)***	4.88 ***(7)***	3.00 ***(2)***	4.90 ***(7)***	4.93 ***(7)***	4.94 ***(7)***
											
Social worker(n = 8)	**4.25 *(6)***	4.01 ***(5)***	4.25 ***(5)***	4.39 ***(6)***	3.01 ***(2)***	4.56 ***(6)***	4.52 ***(6)***	4.50 ***(6)***	4.73 ***(6)***	4.00 ***(5.5)***	4.31 ***(6)***
											
*p value*	***< 0.001***	*< 0.001*	*< 0.001*	*< 0.001*	*< 0.001*	*< 0.001*	*0.003*	*0.014*	*< 0.001*	*< 0.001*	*< 0.001*

***( )^† ^***- competency level was ranked among occupational groups by cluster activities

Among seven professional groups there were significant differences in the total competence level; the lowest to the highest competence levels were for non-qualified support group, Counsellor/Psychologist/Therapist, Occupational therapists, Medical staff, Nurse, Social worker and Psychologist (table 2). The data of the 10 activity clusters showed similar results to those in the total activities, except that psychologists had a lower competency level in Accommodation Support activities (table 2).

The correlation analysis showed no significant association of the averaged competence level with the number of activities engaged in the total (r = -0.03, p = 0.713) and in the 10 clusters excepting a negative relationship in Therapeutic Interventions (r = -0.184, p = 0.028).

Table 3 shows the frequencies of occupational group and other factors and odds ratios (ORs) for the low engagement of total activities. In univariate analysis we found that low engagement was significantly related to non-qualified supporter, counsellor and psychologist. In the multivariate analysis, only the occupational group of psychologist was significantly related to low engagement. The findings from the 10 clusters were similar (table 4). In the activity clusters of Assessment activities, Therapeutic Interventions and Family Support activities, none of those factors was associated with low engagement. In the Monitoring activities, the low engagement was significantly and positively related to non-qualified support workers and psychologists, in the Role Support activities to no clinical training, in the Accommodation activities to psychologist, in the Care Co-ordination to no clinical training, and in the Personal and Professional Care Management Capabilities to psychologists. It was negatively related to Behavioural Family Therapy (BFT) training in the Enabling activities, to social workers in the Role Support Activities, to BFT and protection trainings in the Care Co-ordination activities and to the first degree awarded earlier than 2000 in the Personal and Professional Care Management Capabilities.

**Table 3:** Distribution of occupation and other factors and odds ratio for low engagement of the all activities

	L*		H*										
	
Variable	N	(%)	n	(%)	p	OR	95%	CI	p	**OR**†	95%	CI	p
**Occupational group**													
Community supporter/STR worker/non-qualified nurse	14	18.9	6	7.7	0.001	3.89	1.36	11.11	0.011	2.50	0.57	11.01	0.225
Counsellor/psychotherapist /therapist/medical staff	9	12.2	1	1.3		15.00	1.82	123.8	0.012	3.51	0.25	48.81	0.350
Qualified nurse	33	44.6	55	70.5		1.00				1.00			
Occupational therapists	7	9.5	8	10.3		1.46	0.48	4.39	0.502	1.04	0.22	4.85	0.959
Psychologist	9	12.2	2	2.6		7.50	1.53	36.84	0.013	7.41	1.05	52.38	0.045
Social worker	2	2.7	6	7.7		0.56	0.11	2.91	0.487	0.58	0.09	3.94	0.579
													
**First qualification**													
Undergraduate course	24	36.9	22	29.7	0.117	1.69	0.81	3.56	0.165	1.28	0.41	4.04	0.675
Certificate course	29	44.6	45	60.8		1.00				1.00			
diploma	12	18.5	7	9.5		2.66	0.94	7.55	0.066	2.85	0.78	10.44	0.114
													
**Date of obtaining the first qualification**													
1- 1967 to 1984	11	17.7	14	19.7	0.256	1.09	0.38	3.15	0.877	0.87	0.18	4.11	0.857
2- 1985 to 1999	24	38.7	32	45.1		1.04	0.43	2.52	0.934	0.69	0.20	2.33	0.545
3- 2000 to 2004	13	21.0	18	25.4		1.00				1.00			
4- 2005 to 2010	14	22.6	7	9.9		2.77	0.87	8.78	0.084	2.49	0.61	10.11	0.202
													
**Frequency of Clinical Training**													
0	14	18.7	8	10.3	0.125	1.47	0.52	4.18	0.470	0.24	0.03	2.31	0.216
1-5	25	33.3	21	26.9		1.00				1.00			
6-10	23	30.7	24	30.8		0.81	0.36	1.82	0.602	0.72	0.22	2.34	0.580
11-19	13	17.3	25	32.1		0.44	0.18	1.06	0.067	0.60	0.16	2.22	0.442
													
**Clinical training content**													
Cognitive Behavioural Therapy	16	21.3	19	24.4		1.00				1.00			
Behavioural Family Therapy	4	5.3	15	19.2		0.32	0.09	1.15	0.080	0.23	0.04	1.32	0.100
Trainings on protection	30	40.0	34	43.6		1.05	0.46	2.40	0.912	0.94	0.32	2.72	0.905
Other trainings^‡^	25	33.3	10	12.8	0.004	2.97	1.10	7.99	0.031	3.62	0.50	26.09	0.201
													

L* - low engagement and H*- high engagement.

OR - univariate analysis, OR† - multivariate analysis, including all variables listed in the table and further Clinical Team for adjustment.

^‡ ^Other trainings including Foundation in personal conduct therapy, Effective brief therapy for depression, Cognitive analytical therapy (CAT), Transactional analysis psychotherapy, Emotional transformation therapy (ETT), Systematic family therapy, Transference focussed therapy, Social problem solving therapy for people with personality difficulties, Occupational therapy master module, Mentalisation based therapy (MBT).

**Table 4:** Factors in relation to low engagement in the 10 activities†

Variable	Assessment	Monitoring	Enabling	Therapeutic	Role	Family	Accommodation	Care	Social	Personal
	L* = 78 H* = 75	L* = 79 H* = 74	L* = 116 H* = 37	L* = 73 H* = 80	L* = 77 H* = 76	L* = 76 H* = 77	L* = 78 H* = 75	L* = 80 H* = 73	L* = 76 H* = 77	L* = 75 H* = 78

**Occupational group**										
Community supporter/STR worker/non-qualified nurse		†								
Counsellor/psychotherapist /therapist/medical staff										
Qualified nurse	1.00	1.00	1.00	1.00	1.00	1.00	1.00	1.00	1.00	1.00
Occupational therapists										
Psychologist		†					†			†
Social worker					-					
										
**First qualification**										
Undergraduate course										
Certificate course	1.00	1.00	1.00	1.00	1.00	1.00	1.00	1.00	1.00	1.00
diploma										
										
**Date of obtaining the first qualification**										
1- 1967 to 1984										-
2- 1985 to 1999										-
3- 2000 to 2004	1.00	1.00	1.00	1.00	1.00	1.00	1.00	1.00	1.00	1.00
4- 2005 to 2010										
										
**Frequency of Clinical Training**										
0					†			†		
1-5	1.00	1.00	1.00	1.00	1.00	1.00	1.00	1.00	1.00	1.00
6-10										
11-19										
										
**Clinical training content**										
Other trainings										
Behavioural Family Therapy			-					-		
Cognitive Behavioural Therapy	1.00	1.00	1.00	1.00	1.00	1.00	1.00	1.00	1.00	1.00
Trainings on protection								-		
										

L* - low engagement and H*- high engagement.

† all data analysis, results and table note were similar to those in table 3. Thus, we only presented these statistical significances in

the associations in the table; "†" means a significantly positive association, "-" a negative association.

Table 5 shows the frequencies of occupational group and other factors and ORs for the low competence of total activities. Compared to qualified nurses, non-qualified supporters had a significant low competence, and psychologists had a high competence. The low competence was also significantly increased with later time of obtaining first qualification and CBT training. It was not related to the type of first qualification. In the multivariate analysis, the associations were similar to those in the univariate analysis, except for no significance for psychologist and BFT training (table 5).

**Table 5:** Distribution of occupation and other factors and odds ratio for low competence of the all activities

	L*		H*										
	
Variable	n	(%)	n	(%)	p	OR	95%	CI	p	OR†	95%	CI	p
**Occupational group**													
Community supporter/STR worker/non-qualified nurse	17	22.4	3	3.9		6.80	1.86	24.88	0.004	12.56	1.63	96.52	0.015
Counsellor/psychotherapist /therapist/medical staff	6	7.9	4	5.3		1.80	0.47	6.83	0.387	2.60	0.27	24.78	0.407
Qualified nurse	40	52.6	48	63.2		1.00				1.00			
Occupational therapists	9	11.8	6	7.9		1.80	0.59	5.49	0.301	2.16	0.43	10.98	0.353
Psychologist	1	1.3	10	13.2		0.12	0.01	0.98	0.048	0.57	0.04	9.24	0.694
Social worker	3	3.9	5	6.6	0.002	0.72	0.16	3.20	0.666	0.38	0.04	3.39	0.384
													
**First qualification**													
Undergraduate course	25	38.5	21	28.4		1.40	0.67	2.93	0.371	3.51	0.84	14.67	0.086
Certificate course	34	52.3	40	54.1		1.00				1.00			
diploma	6	9.2	13	17.6	0.241	0.54	0.19	1.58	0.263	0.39	0.10	1.56	0.182
													
**Date of obtaining the first qualification**													
1- 1967 to 1984	4	6.5	21	29.6		0.23	0.06	0.83	0.025	0.32	0.05	2.07	0.232
2- 1985 to 1999	28	45.2	28	39.4		1.21	0.50	2.93	0.665	1.95	0.48	7.89	0.349
3- 2000 to 2004	14	22.6	17	23.9		1.00				1.00			
4- 2005 to 2010	16	25.8	5	7	0.001	3.89	1.14	13.27	0.030	15.76	2.18	113.9	0.006
													
**Frequency of Clinical Training**													
0	8	10.5	14	18.2		0.74	0.26	2.11	0.578	4.88	0.52	45.83	0.165
1-5	20	26.3	26	33.8		1.00				1.00			
6-10	25	32.9	22	28.6		1.48	0.65	3.35	0.349	5.30	1.19	23.60	0.029
11-19	23	30.3	15	19.5	0.232	1.99	0.83	4.77	0.122	12.67	2.13	75.40	0.005
													
**Clinical training content**													
Cognitive Behavioural Therapy	23	30.3	12	15.6		1.00				1.00			
Behavioural Family Therapy	6	7.9	13	16.9		0.24	0.07	0.79	0.019	0.27	0.05	1.53	0.14
Trainings on protection	34	44.7	30	39	0.035	0.59	0.25	1.39	0.228	0.64	0.18	2.25	0.484
Other trainings^‡^	13	17.1	22	28.6		0.31	0.12	0.82	0.018	0.15	0.02	1.00	0.05

L* - low competence and H*- high competence.

OR - univariate analysis, OR† - multivariate analysis, including all variables listed in the table and further Clinical Team and number of engagement activities (tertile) for adjustment.

^‡ ^Other trainings including Foundation in personal conduct therapy, Effective brief therapy for depression, Cognitive analytical therapy (CAT), Transactional analysis psychotherapy, Emotional transformation therapy (ETT), Systematic family therapy, Transference focussed therapy, Social problem solving therapy for people with personality difficulties, Occupational therapy master module, Mentalisation based therapy (MBT).

The multivariate analysis for the 10 clusters showed similar results to those in the total activities (table 6). Further analysis of the 47 subheading clustering data showed similar results, but less statistical significances (data on request).

**Table 6:** Factors in relation to low competence in the 10 clusters activities

Variable	Assessment	Monitoring	Enabling	Therapeutic	Role	Family	Accommodation	Care	Social	Personal
**Occupational group**										
Community supporter/STR worker/non-qualified nurse	†		†	†		†			†	
Counsellor/psychotherapist /therapist/medical staff										
Qualified nurse	1.00	1.00	1.00	1.00	1.00	1.00	1.00	1.00	1.00	1.00
Occupational therapists										
Psychologist										
Social worker										
**First qualification**										
Undergraduate course										
Certificate course	1.00	1.00	1.00	1.00	1.00	1.00	1.00	1.00	1.00	1.00
diploma										
**Date of obtaining the first qualification**										
1- 1967 to 1984				-	-					
2- 1985 to 1999										
3- 2000 to 2004										
4- 2005 to 2010	†	†	†			†		†	†	
**Frequency of Clinical Training**										
0										
1-5	1.00	1.00	1.00	1.00	1.00	1.00	1.00	1.00	1.00	1.00
6-10			†							
11-19	†		†							
**Clinical training content**										
Other trainings							-			
Behavioural Family Therapy										
Cognitive Behavioural Therapy	1.00	1.00	1.00	1.00	1.00	1.00	1.00	1.00	1.00	1.00
Trainings on protection							-			

† all data analysis, results and table note were similar to those in table 3. Thus, we only presented these statistical significances in the associations in the table; "†" means a significantly positive association, "-" a negative association.

## Discussion

In this NHS community-based mental health workforce study, we found that *Monitoring activities*, *Assessment activities *and *Role support activities *- *informal counselling *were the most common activities, while *Role support activities - social support and therapeutic interventions *were less commonly engaged. Although this was across all groups, one would have expected there to be a higher level of engagement in therapeutic interventions. The overall competency level was reasonably good and qualified nurses had an average competency level of 'proficient'. But the competence level varied with professional groups across 10 clusters activities. Low competence level was associated with non-qualified community support professions, late time of obtaining a first qualification, more frequent clinical training, and CBT training.

In an Irish national survey of client care activities carried out by community psychiatric nurses, McCardle et al [7] observed that the common client care activities engaged in by their sample were assessment activities, listening, medication management (including compliance and education), practical support for daily living activities and providing family support while delivery of cognitive behavioural therapies and structured family therapies were limited. These were similar to our findings. Our data expands upon the work of McCardle et al. [7] by examining engagement in therapeutic activities across multidisciplinary teams. Surveys looking at multidisciplinary teams [8] showed that community mental health teams as a whole engaged in assessment activities and supporting carers and family, which were the same as our data, but their study suggested that the most common activity was the provision of therapy or counselling. The variation may be due to differences in mental health workforce staff between the two studies or the fact that the present study looked at the services provided by each mental health team rather than individual staff members' activities.

To our knowledge, our survey is the first study showing competency levels and their determinants for clinical activities in community-setting mental health workforce within the UK. A study conducted by Greaves et al [9] in Australia found self-reported competence levels of occupational therapists working in mental health settings to be comparable to other occupational groups, and significantly higher in community settings, which is consistent with our data in England. Previous studies in general clinical settings [10] have also provided some insight as to the self reported competence levels of non-qualified support staff, and suggested that the changing roles of this occupational group mean that they are increasingly taking on more advanced clinical responsibilities. As a result ongoing supervision of this group is required to maximise the contribution they can make to patient care [11]. As we move into an era of large scale workforce reductions in the NHS, the need to develop cost effective roles to deliver parts of the service will become more evident.

Previous studies [12] have indicated that increased length of professional experience in health care settings was not necessarily indicative of improved performance. But our data, particularly with multivariate adjustment analysis, suggested that those who were more recently qualified may have lower competency levels than those who had a number of years with previous experience in practice. Gauntlett [13] found that a lack of opportunities to practice and training at a level that is too basic, may result in insufficient levels of competence being obtained, particularly for more specialised therapeutic interventions. Our findings that not all clinical training courses, nor a high frequency of training courses undertaken by staff were related to increased competency levels echo Gauntlett's [13] assertion that post-graduate mental health training programmes need greater evaluation to ensure their effectiveness. By aligning service provision along the care pathways, there would be scope to ensure that sufficient ratios of staff are trained to advanced levels on specific therapeutic intervention modalities. Knowing where the specialist skills lie will be key in managing workload when allocating clients to pathways to match supply and demand.

The main contribution of this survey lies in what it tells us about the competency level and its determinants in a representative community mental health workforce in England. The Trust included various professional groups. Its size and advanced state of development in delivering care based on the YCPM meant that it was the most appropriate site for the purposes of the study and that the findings would be sufficiently robust to be indicative of the general state of readiness and education needs of the workforce of large NHS Mental Health Trusts with high community and outreach services, seeking to deliver care using this model. A second strength is that we carried out a standardised YCPM questionnaire and used a multivariate regression model to analyse the data. Our study has limitations. (1) The survey response rate was relatively low, in comparison with other surveys that have been conducted in the NHS setting [14]; this may be attributable to high staff workloads, but the response rate is similar to that in our previous questionnaire study in the community in the UK, where there seemed no significant biases introduced [15,16]. (2) This was a service evaluation focused on staff perceptions and as such the engagement and competence levels were self-reported by participants in the questionnaire. This may lead to the well-reported social desirability effect [17] and therefore caution should be paid to the reported high level of competence, but it would not necessarily affect findings of low competences in given activities where further training is recommended. The current findings are supported by the focus groups data. Thus the low competence for occupational group and its determinants were ensured. (3) Caution should be exercised in interpreting the causal-result association for the determinants of low competency because the study was cross-sectional. (4) Our studied population is a sample of different professions in the English health care system and caution should be exercised in applying our findings in other mental health systems.

*Summary and recommendation*, our study has succeeded in profiling the intervention activities engagement and competency levels in the community setting mental health workforce in England. The findings serve as an indicator of the competencies and educational needs of wider Mental Health workforce of the region and nation. However, the findings are based on self-reported levels of competency and given this limitation, we recommend that further studies using this approach would benefit from a more structured use of an expert reference group to evaluate and comment on findings. As identified in *No health without mental health *[18], services will have £400 million invested over the next four years to make a choice of psychological therapies available for those who need them, expanding provision to most disadvantaged groups. As we move into a new era of Payment by Results (PBR) in mental health, there will inevitably be a sharper focus upon therapeutic interventions and outcomes. This review of current therapeutic activities and interventions will help to ensure that the mental health workforce is sufficiently equipped with the required level and depth of skills. Our findings further indicate a number of ways in which mental health provider organisations may wish to utilise the competency survey, for example where pathways have been aligned to clinical teams to identify areas where skills and competencies may require strengthening. Alternatively the survey could be used to identify where teams could be realigned or reconfigured to provide care along care pathway structures.

The continuing development of the mental health curriculum means that training needs and competency levels will apply to the current workforce, and may have limited generalisability to the workforce being currently trained and qualifying in the near future.

## Conclusions

This research has suggested that the self rating competency based questionnaire approach utilised could inform the evaluation and redesign of roles within mental health teams, which is required when implementing a new model of care such as the Yorkshire Care Pathway Model as described here. Within the limits of this study, our findings indicate that mental health staff in this community-setting felt competent to perform most activities itemised in the YCPM. Variations in competence both within and across professional groups in relation to cluster activities can inform the development of bespoke training programmes and offer opportunities to model team competency requirements that are not allied to specific disciplines, supporting the value of multidisciplinary team working. Our survey's results indicate the need for more effective rather than more frequent in-service training to support staff development, in particular more targeted training where there is increasing service demand for example in the provision of certain behavioural therapies.

## Competing interests

The authors declare that they have no competing interests.

## Authors' contributions

LL generated the research ideas and participated in the design of the study, and interpreted the findings and drafted the manuscript. SO carried out data collection, performed statistical analysis and drafted the manuscript. DS participated in the conception, design and coordination of the study, helped in data collection and data analysis. THM generated the research ideas and participated in the design and coordination of the study, helped to draft and reviewed the manuscript. DMZ analysed the data, interpreted the study findings and had critical comments on the manuscript. SC participated in the conception, design and coordination of the study, helped in data collection and data analysis. SD participated in the design and coordination of the study, and reviewed the manuscript. BB participated in the design of the study, and reviewed the manuscript. DB participated in the design of the study, and reviewed the manuscript. LM carried out the literature review and commented on the manuscript. RC participated in the conception and design and coordination of the study, supervised data analysis, interpreted the findings and drafted the manuscript and revised the manuscript. All authors read and approved the final manuscript.

## Pre-publication history

The pre-publication history for this paper can be accessed here:

http://www.biomedcentral.com/1472-6963/11/352/prepub
